# Cytotoxicity of *Atriplex confertifolia*

**DOI:** 10.1155/2010/976548

**Published:** 2010-03-16

**Authors:** Christopher J. Capua, Nick P. Hopson, C. Malcolm M. Stewart, G. Robert Johnston, Kim L. O'Neill, G. Bruce Schaalje, Christopher M. Lee, Gary M. Booth

**Affiliations:** ^1^Department of Plant and Wildlife Science, Brigham Young University, 275 Widtsoe Building, Provo, UT 84602, USA; ^2^Department of Microbiology and Molecular Biology, Brigham Young University, 775 Widtsoe Building, Provo, UT 84602, USA; ^3^Department of Statistics, Brigham Young University, 230 Talmage Building, Provo, UT 84602, USA; ^4^Department of Radiation Oncology, Cancer Care NW, 601 S. Sherman Avenue, Spokane, WA 99202, USA

## Abstract

The search for cancer treatment continues to be a global effort. As part of this global effort, many natural products have been tested against cancer cell lines, mostly from tropically located plants. This study reports that extracts of *Atriplex confertifolia* (Torr. and Frem.) S. Watson (Chenopodiaceae), a native North American plant (also known as shadscale or saltbush), has significant bioactivity against human breast cancer cell lines MCF-7, MDA-MB 435, MDA-MB 231, and HeLa cells (cervical cancer cells). The bioactivity of *A. confertifolia* extracts on these cells lines was compared to an FDA-approved cancer drug (Onxol^®^) and an industry-standard leukocyte control cell line. Active portions of the extracts were found primarily in the polar fractions of the plant. A dose-response curve of the extracts displayed significant cell death similar to Onxol^®^. The plant extracts did not significantly inhibit the viability of the leukocyte cell line. In a timed study, over 90% of cell lines MDA-MB 435 and HeLa died after 24 hours. Cell death appears to result from apoptosis.

## 1. Introduction

In 2008, 565,000 Americans died of cancer. Cancer is the second most common cause of death in the US, exceeded only by heart disease [1]. Pollner [2] reported that cancer in the United States has more than doubled in the last 30 years, from 1 in 20 in 1960 to 1 in 9 today. Although cancer is not the number one cause of mortality in the United States it is often painful, and is the most feared of diseases [3]. Therefore, the search for cancer treatments will continue until a cure is found.

In the past 10 years new technology has provided additional therapies. For example, imaging technology has delivered tomosynthesis [4] and advances in genetics have produced a variety of antiangiogenesis drugs. However, as Bettelheim [5] noted, “The war on cancer isn't just fought with bioengineered drugs and souped-up genes. Scientists also utilize ornamental shrubs, tree bark, sea horses, and thousands of other natural products that serve as the basis for new cancer drugs.”

 Each year thousands of plant extracts are screened for bioactivity against cancer [5]. Most botanical investigations have come from rainforest or tropical plants, yet there are many untested nontropical plants and a few have shown bioactivity [6, 7]. One such example is Taxol, the number one selling cancer drug, which is derived from Pacific yew tree bark. Though, its initial discovery was not enthusiastically endorsed by the medical community, its success has precipitated an intensive search for new natural product treatments. Other plant-derived drugs that have been discovered include topotecan, vincristine, and vinblastine [5]. 

A Brigham Young University (BYU) study was completed in 2001 to screen more than 40 plants of North America for their cytotoxicity [8]. One of the plants, *Atriplex confertifolia *(Torr. and Frem.), S. Watson (Chenopodiaceae), showed much greater bioactivity than others. This plant (also known as shadscale or saltbush) is widely distributed throughout North America from Texas to North Dakota and west to the Pacific Ocean. The majority of studies on *A. confertifolia* have been focused on its distribution [9], lifespan [10], botanical and ecological characteristics [11] and how it has been affected by grazing [12]. No studies had been performed on the bioactivity of *A. confertifolia* until 2004, when Welch [13] determined the cytotoxicity effects of *A. confertifolia* on human cervical cancer cells (HeLa cells).

*A. confertifolia* is known to provide a source of palatable, nutritious forage for a wide variety of wildlife and livestock. Specifically, the fruits and leaves are a food source for deer, desert bighorn sheep, pronghorn, small rodents, jackrabbits, game birds, and songbirds [14]. Of all *Atriplex* genera in North America, *A. confertifolia* is ecologically the most important and can grow on a greater variety of soils. Welch [13] found that the most bioactive fraction of *A. confertifolia* killed more than 94% of the HeLa cells in a laboratory bioassay. “The fact that *A. confertifolia* is edible but still kills cancer cells may be very important. It suggests that the cytotoxic agents in the plant may show specificity only towards cancerous cells, making it an excellent candidate for pharmacological use [13].” From the positive cytotoxicity results of Welch's study using HeLa cells, it was thought that *A. confertifolia* may have bioactivity on other human cancer cells.

## 2. Materials and Methods

### 2.1. Source of Plant Material

All procedures used the same samples of *Atriplex confertifolia* that were taken west of Lehi, Utah (40° 13′ 51′′ N, 112° 11′ 33′′ W) and stored at 4°C in a cold room at Brigham Young University (BYU). Collection of *Atriplex confertifolia *(Torr. and Frem.) S. Watson (Chenopodiaceae) was performed by Gary M. Booth on July 2004 as voucher specimen number 0001. This plant was deposited as a voucher specimen at the Stanley L. Welch Herbarium at Brigham Young University and identification was confirmed by curators at the herbarium.

### 2.2. Isolation

#### 2.2.1. Extraction and Bilayer Separation

The leaves, stems, and branches of *A. confertifolia *were cut or chopped into 2.5 cm or smaller pieces and then further homogenized using a common mortar and pestle. Approximately 23 g of crude, dry plant material were added to a 250 mL Erlenmeyer Flask. Then 130 mL of methanol were added to the flask and the mixture was stirred on a stir plate for 24 hours. This methanol solution was then filtered using Whatman No. 30 (11.0 cm) filter paper and the supernatant was retained. 

Approximately 3 mL of the methanol/*Atriplex* extraction were placed into a 15 mL screw-cap conical test tube. This was followed by the addition of 3 mL of distilled water to the test tube and then 3 mL of hexane. The test tube was then tightly capped and shaken vigorously for 20 seconds. This was usually done with a sequence of four test tubes at a time. These test tubes were then centrifuged for 5 minutes at 1500 rpm. The polar methanol/water portions dissolved the polar compounds, while hexane dissolved the nonpolar compounds, resulting in an aqueous hexane bi-layer. The hexane fraction formed the upper phase in the test tube. The hexane was then pipetted from the methanol /water portion using a standard Pasteur pipette. 

#### 2.2.2. Cell Culture Lines

The following cell lines were obtained from ATCC The Global Bioresource Center [15] and were used in the current study: ATCC number HTB-22 designation of MCF-7 was established from pleural effusion from a 69-year-old female with adenocarcinoma [16]. MCF-7 cells were recommended as target cells because of their widely acknowledged estrogen sensitivity [17]. ATCC number HTB-26 designation of 231, adenocarcinoma of human breast mammary gland was obtained from a 51-year-old female. MD Anderson number MDA-MB designation of 435, adenocarcinoma of human breast mammary gland was obtained from a 31-year-old female. ATCC number CCL-2 designation of HeLa was an adenocarcinoma epithelial cell of the cervix obtained from a 31-year-old female [15]. Finally, normal human lymphocyte cells, specifically monocytes, from a healthy 28-year-old male were used as a control. Population doubling times for the MCF-7, MDA-MB 435, MDA-MB 231, and HeLa cell lines were estimated at 36, 16, 24–36, and ~80 hours, respectively [18–20].

#### 2.2.3. Nonpolar and Polar Extracts

A bioassay was then performed using each cell line to determine which portion showed cytotoxicity. The bioassay was performed in the following manner:

a 2 mL volume of the methanol/water portion was added to a 2 mL Eppendorf tube (microcentrifuge tube) and 2 mL of the hexane were added to another 2 mL Eppendorf tube. These tubes were allowed to evaporate to dryness. A volume of 300 *μ*L of Roswell Park Memorial Institute 1640 (RPMI1640), which is the cell growth medium, was then added to each Eppendorf tube. These tubes were then capped and thoroughly mixed using a sonicator (Cole-Parmer 8851) and a deluxe mixer (Scientific Products).

A volume of 40 *μ*L of each sample was then added to each of three wells in a 96-well flat-bottom plate that was prepared with a cell solution with a concentration of 0.8–1 × 10^5^ cells/mL. Volumes of 50 *μ*L each of RPMI1640 were also added to a total of 9 wells in the plate to serve as controls. The plate was allowed to incubate for 24 hours and then stained with a sulforhodamine stain. 

Living cells were stained while dead cells were washed away. Once the cells were stained they were read using a BioTek EL800 spectrofluorometer at 570 nm. The number of viable cells in the control wells was then compared to the number in the wells treated with the methanol/water and with the hexane portions of the *A. confertifolia* extraction/separation. The cytotoxic fraction was then detected by finding which portion showed the lowest cell viability. 

#### 2.2.4. Dose-Response

The dose response curve was obtained by the following procedure: 

once the fraction from the column that showed the highest degree of cytotoxicity was identified using the cell bioassay described in the isolation procedure above, it was placed in a pre-weighed 2 mL Eppendorf tube and allowed to evaporate. Small portions of the cytotoxic fraction were then added to the Eppendorf tube and allowed to evaporate in this fashion until approximately 9 mg of the *A. confertifolia* extract were dried in the bottom of the Eppendorf tube. 

A total of 1 mL of RPMI1640 was added to the completely dry *A. confertifolia* extract in the Eppendorf tube and thoroughly mixed with a sonicator (Cole-Parmer 8851) and a deluxe mixer (Scientific Products) so that all of the dry *A. confertifolia* extract was in solution, resulting in an *A. confertifolia* concentration of 9 mg/mL. Volumes of 50 *μ*L of normal RPMI1640 was then added to three wells in a previously prepared 96-well flat-bottomed plate where each well had 150 *μ*L of a 0.8–1 × 10^5^ cells/mL solution. This represented a dosage of 0 mg per mL. Normal RPMI1640 were also added to 9 other wells on the plate as the control for the experiment. 

A total of 45 *μ*L of the normal RPMI1640 was then added to three wells of the plate. To these same three wells, 5 *μ*L of the treated RPMI1640 were added. This gave these three wells a total concentration of 0.23 mg/mL. The calculated concentration takes into account that 150 *μ*L of RPMI1640 had been added to each well when the cells were originally added to the plate. Thus, the total liquid volume in each well was now 200 *μ*L. In the next three wells, 40 *μ*L of normal RPMI1640 were added, and 10 *μ*L of the treated RPMI1640 was also added to create a concentration of 0.46 mg/mL.

This pattern was continued until 50 *μ*L of treated RPMI1640 was placed in each of three wells with no normal RPMI1640. Those wells resulted in a concentration of 2.28 mg/mL. Three more wells containing 45 *μ*L of the normal RPMI1640 were prepared. To these same three wells, 5 *μ*L of diluted treated RPMI1640 were added. This step was continued until concentrations of 0.12, 0.06, and 0.03 mg/mL were obtained. This 96-well plate was then incubated for 24 hours and then subjected to the sulforhodamine staining procedure so that the viability of the cells could be measured. These data were then plotted graphically as dose-response curves. The data were transformed to the log scale and analyzed using a linear mixed model program [21]. A first-order model, second-order model and a separate means model were then fitted. 

#### 2.2.5. FDA-Approved Drug Comparison

Dose-response curves from the *A. confertifolia *extracts were then compared to the chemotherapy drug Onxol^®^ dose response curves obtained by the following procedure:

Onxol^®^ drug comes in liquid form at a concentration of 4 mg/mL. Volumes of 50 *μ*L of normal RPMI1640 were then added to three wells in a previously prepared 96-well flat-bottomed plate where each well had 150 *μ*L of a 0.8-1x10^5^ cells/mL solution. This represented a control dosage of 0 mg/mL. Normal RPMI1640 was also added to 9 other wells on the plate as another control for the experiment. 

 A total of 45 *μ*L of the normal RPMI1640 was added to three wells of the plate. To these same three wells, 5 *μ*L of the Onxol^®^ were added. This gave these three wells a total concentration of 0.15 mg/mL. The calculated concentration takes into account that 150 *μ*L of RPMI1640 had been added to each well when the cells were originally added to the plate. The total liquid volume in each well was now 200 *μ*L.

A total of 40 *μ*L of normal RPMI1640 were added to the next three wells, and then 10 *μ*L of the Onxol^®^ was added to create a concentration of 0.30 mg/mL. This pattern was continued until 50 *μ*L of Onxol^®^ were placed in each of three wells with no normal RPMI1640. This gave those wells a concentration of 1.52 mg/mL. 

To three more wells, 45 *μ*L of the normal RPMI1640 were added. To these same three wells, 5 *μ*L of diluted Onxol^®^ were added. This step was continued until concentrations of 0.08, 0.04, and 0.02 mg/mL were obtained. This 96-well plate was then incubated for 24 hours and subjected to the sulforhodamine staining procedure so that the viability of the cells could be measured. 

#### 2.2.6. Timed Response

The timed response curve was obtained by the following procedure: 

once the fraction from the column that showed the highest degree of cytotoxicity was identified using the cell bioassay described in the isolation procedure above, it was placed in a pre-weighed 2 mL Eppendorf tube and allowed to evaporate. Small portions of the cytotoxic fraction were then added to the Eppendorf tube and allowed to evaporate in this fashion until approximately 9 mg of the *A. confertifolia* extract were dried in the bottom of the Eppendorf tube. 

A total of 1 mL of RPMI1640 was added to the completely dry *A. confertifolia* extract in the Eppendorf tube and thoroughly mixed with a sonicator (Cole-Parmer 8851) and a deluxe mixer (Scientific Products) so that all of the dry *A. confertifolia* extract was in solution, resulting in an *A. confertifolia* concentration of 9 mg/mL. Volumes of 50 *μ*L of normal RPMI1640 were then added to three wells in a previously prepared 96-well flat-bottomed plate where each well had 150 *μ*L of a 0.8–1 × 10^5^ cells/mL solution. This represented a dosage of 0 mg per mL. Normal RPMI1640 was also added to 9 other wells on the plate as the control for the experiment. 

Volumes of 15 *μ*L of the normal RPMI1640 were added to three wells of the plate. To these same three wells, 35 *μ*L of the treated RPMI1640 were added. This gave these three wells a total concentration of 1.59 mg/mL. The 96-well plate was then incubated for 1 hour and then subjected to the sulforhodamine staining procedure. Another 96-well plate that had been prepared in the same fashion was incubated for 2 hours and then subjected to the sulforhodamine staining procedure. Also a third 96-well plate prepared in a similar manner was incubated for 4 hours and then subjected to the sulforhodamine staining procedure. This procedure was continued at increments of 2 hours up to 24 hours from the time the first treated RPMI1640 was added. These data were then plotted graphically as time-response curves. The data were transformed to the log scale and analyzed using a linear mixed model program [21]. A first-order model was then used to fit the data.

#### 2.2.7. Cell Preparation for Scanning Electron Microscopy

The scanning electron microscopy images were obtained by the following procedure:

once the fraction from the column that showed the highest degree of cytotoxicity was identified using the cell bioassay described in the isolation procedure above, it was placed in a pre-weighed 2 mL Eppendorf tube and allowed to evaporate. Small portions of the cytotoxic fraction were then added to the Eppendorf tube and allowed to evaporate in this fashion until approximately 9 mg of the *A. confertifolia* extract were dried in the bottom of the Eppendorf tube. 

A total of 1 mL of RPMI1640 was added to the completely dry *A. confertifolia* extract in the Eppendorf tube and thoroughly mixed with a sonicator (Cole-Parmer 8851) and a deluxe mixer (Scientific Products) so that all of the dry *A. confertifolia* extract was in solution, resulting in an *A. confertifolia* concentration of 9 mg/mL. Volumes of 3 mL of solution of 0.8–1 × 10^5^ cells/mL were pipetted onto on each of two microscope slides. A total of 1 mL of normal RPMI1640 was added to one slide as a control for the experiment, and one mL of treated RPMI1640 was added to the other slide. These were left to incubate for 6–8 hours. The slides were then subjected to a routine SEM preparation.

HeLa cells were prepared for imaging using a Scanning Electron Microscope (SEM) model Philips XL30 ESEM FEG located at the Cluff Building, BYU.

## 3. Results and Discussion

**Figure 1:**
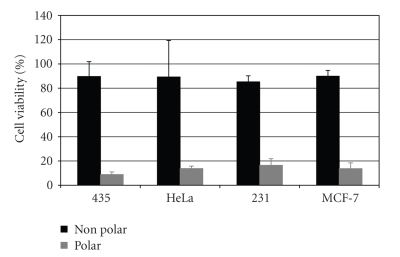
Bioactivity of the nonpolar and polar extracts of *Atriplex confertifolia *against three human breast cancer cell lines (435, 231, and MCF-7) and a human cervical cancer cell line (HeLa).

*Atriplex confertifolia* was first shown to contain bioactive compounds during a cooperative study between BYU and the New York Botanical Garden [13]. Figure 1 shows in a similar manner that the bioactive component(s) of the *A. confertifolia* are found primarily in the polar methanol/water portion of the extraction. The polar fraction killed about 90% of the cells on all cell lines, while the nonpolar hexane fraction only reduced cell viability by less than 20%.

These results are similar to the study reported by Welch [13], but are in contrast to the work done by Donaldson [22] who, while doing HeLa cell bioassays, found that *Atriplex canescens* showed activity against HeLa cells with hexane fractions (48.7% cell inhibition), but no activity with methanol fractions (0.9% cell inhibition). However, Davis [23] clearly demonstrated that methanol fractions were more toxic than hexane fractions when tested against a wide variety of plants on HeLa cells and 3T3 fibroblasts as a control line. 

**Figure 2:**
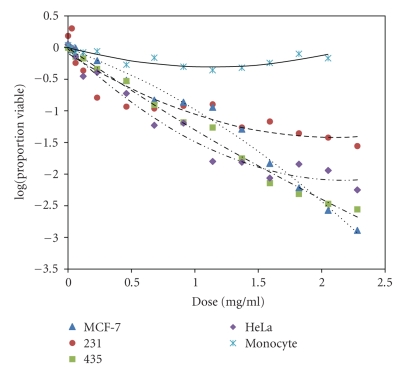
Comparison of the dose-response curves for extracts of *Atriplex confertifolia *against three human breast cancer cell lines; 435, 231, MCF-7, HeLa, and normal human lymphocyte (monocyte).

When the *A. confertifolia *extracts from the active fraction were administered at different concentrations to the cell lines, cell viability showed a dose-response. The doses ranged from 0.03 mg/mL to 2.28 mg/mL. Cancer cell viability ranged, on average, from 95 to ≤10% after exposing the cell lines to varying concentrations of the *A. confertifolia *compounds for 24 hours. The extract was apparently highly selective since the monocyte control cells were affected very little by the extract (Figure 2). Comparing full and 2nd-order log linear data models by a lack of fit test gives a *χ* ^2^ = 12.1 and a *P *≤ .001, demonstrating the 2nd-order log linear model is a preferred model. Similar data were found comparing 1st-order log linear and 2nd-order log linear *χ* ^2^ = 63.3 and a *P* ≤ .05.

Overall there were significant differences among the curves (*F* = 16.97, *P* ≤ .0001). Among the four cancer cell lines, there were also significant differences for all pairs of lines, with the smallest *F* = 5.50, and largest *P* = .0043. These dose-response curves are similar to those reported by Lau et al. [24], who used multiple pancreatic adenocarcinoma cell lines to study the anticancer effects of the fruit *Brucea javanica*. They reported a dose range of 0.1 *μ*g/mL to 1000 *μ*g/mL and a cell survival rate of 90% to 20% after exposing the cells to the active compound for 72 hours. Medina-Hoguín [25] showed that root oils of a desert plant *Anemopsis californica* had antiproliferative activity against AN3CA and HeLa cells in vitro but no activity against lung, breast, prostate or colon cancer cells. Hence, *A. confertifolia *also showed activity against a variety of cell lines.

After the dose reached a concentration of approximately 1.59 mg/mL, the cell viability leveled off at approximately 10%. This plateau is seen in other dose-responses listed in the literature also. For example, the dose-response curves reported by Sadeghi-Aliabadi and Ahmadi [26] started to plateau at about 20% cell viability. 

**Figure 3:**
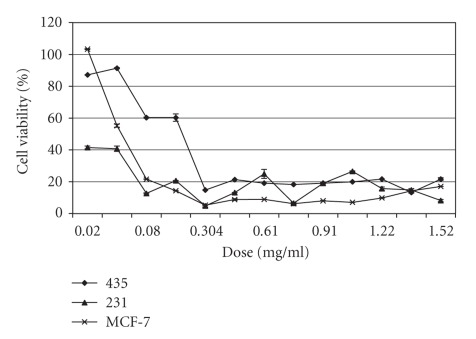
Toxicity of Onxol^®^ to human breast cancer cell lines 435, 231, and MCF-7.

The toxicity data from the *A. confertifolia* extracts (Figure 2) are similar to the dose-response curves generated using the chemotherapeutic drug Onxol^®^ (Paclitaxel) (Figure 3). These data clearly show Onxol^®^ has excellent cytotoxicity, with a significant dose response (*F* = 8.51, *p* < .0001) that differed among cell lines (*F* = 1.71, *P* = .0315). For Onxol^®^, lower concentrations (0.3 mg/mL) cause near 100% mortality. Gangadevi and Muthumary [27] have shown similar findings, wherein Paclitaxel caused about 80% apoptosis in each of five cell lines. Although the *A. confertifolia* extract did not elicit the exact same dose-response curves, it did show a similar degree of toxicity in the higher doses as shown in Figure 2. 

We may also take into consideration that extracts of *A. confertifolia* have not been purified to single compounds, which are causing cell death. With future isolation procedures a more potent concentration of *A. confertifolia* might be obtained resulting in even higher levels of toxicity at even lower doses than the currently FDA-approved drugs.

**Figure 4:**
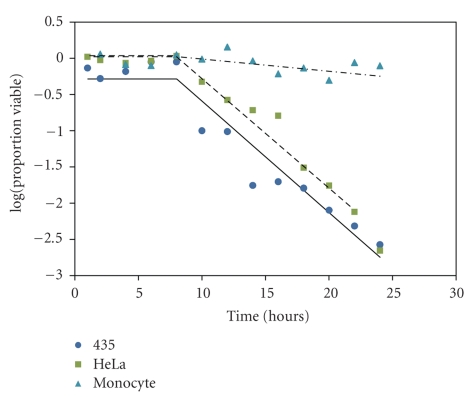
Timed toxicity response of cell lines 435, HeLa, and monocyte control to 2.05 mg/mL of extract from *Atriplex confertifolia*.

To examine cell viability over time, a dose of 2.05 mg/mL was administered to a breast cancer cell line (435), a cervical cancer cell line (HeLa), and the control monocyte cell line. This dose was chosen because it typically demonstrated over 90% mortality. These cultures were allowed to incubate from 1 to 24 hours. After 8 hours of incubation, cancer cell viability decreased precipitously especially for the breast cancer cells (Figure 4). Again, monocyte control cells did not appear to be greatly affected. After 18 hours of incubation, both cancer cell cultures were reduced to approximately 20% viability. There were significant differences among all three curves (*F* = 168.89, *P* < .0001). However, there is no statistical difference between the cancer cell lines (*T* = −0.9, *P* = .93). 

 Many chemotherapy drugs, such as Paclitaxel and colchicines, interfere with the normal function of microtubule breakdown. Colchicine causes the depolymerization of microtubules whereas Paclitaxel arrests their function by having the opposite effect; it hyper-stabilizes their structure. This destroys the cell's ability to use its cytoskeleton in a flexible manner and does not have the ability to disassemble. This adversely affects cell function because the shortening and lengthening of microtubules (termed dynamic instability) is necessary for their function as a mechanism to transport other cellular components [28]. It is possible that *A. confertifolia *could be acting in a similar manner. However, only additional experimentation could demonstrate that affect.

The delay in toxicity shown in Figure 4 suggests that it takes approximately 8 hours for the toxic compounds to enter the cell thus resulting in death either by apoptosis or necrosis. Apoptosis is controlled cell death while necrosis is the immediate complete breakdown of the plasma membrane, resulting in the release of intercellular proinflammatory molecules [29]. 

**Figure 5:**
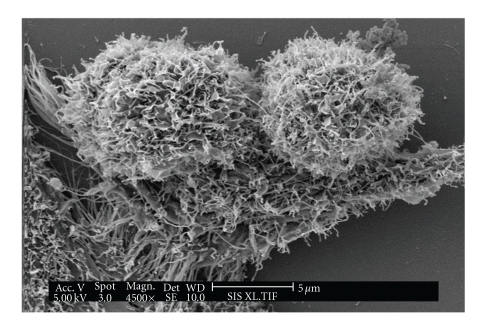
Scanning electron micrograph (SEM) of two normal HeLa cancer cells.

**Figure 6:**
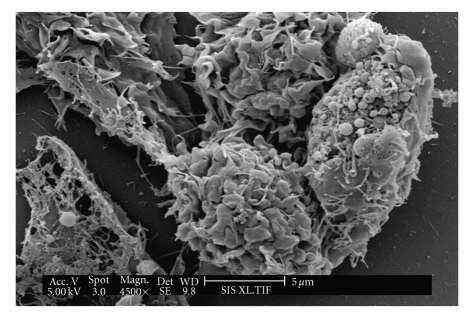
Scanning electron micrograph of a HeLa cell treated with extracts of *Atriplex confertifolia* showing the formation of apoptotic bodies.

Figure 5 shows an SEM of two normal HeLa cells, while Figure 6 shows a HeLa cell treated with 2.05 mg/mL of *A. confertifolia* for 6–8 hours. The contorted-looking state of the treated cell in Figure 6 shows cellular blebbing and the formation of apoptotic bodies. The blebbing is an irregular bulge in the plasma membrane typical of a cell undergoing apoptosis. These bulges noted in Figure 6 often separate from the cell, taking a portion of the cytoplasm with them [29, 30]. Hence, these data suggest that *A. confertifolia *kills HeLa cells by apoptosis and not by necrosis.

In conclusion, our publication data suggests that extracts of *A. confertifolia *may cause cell death in three types of breast cancer cells and a cervical cancer cell line (HeLa), but does not affect monocyte control cells. It is clear that the majority of toxic compounds are found in the polar fractions of this plant extract. The toxicity of *A. confertifolia *and the concentration of the extracts were generally comparable to those of the FDA-approved drug Onxol^®^ especially at the highest doses, although Onxol^®^ killed the cells more completely at lower doses. In addition, these data also show it takes approximately 8–10 hours before cell mortality can be detected. This was observed for both breast and cervical cancer cells. The monocyte control culture was again not affected by the 24 hours incubation period with *A. confertifolia*. Thus it is clear that extracts of *A. confertifolia* causes cell death via apoptosis and not by necrosis. *A. confertifolia* is one of the few nontropical desert species tested to date that shows selective bioactivity against a variety of human cancer cells.
